# Sixty Thousand Pounds for the London Hospitals

**Published:** 1895-10-26

**Authors:** 


The Hospital

An Institutional Journal of

The Medical Sciences and Hospital Administration, with

Vol. XIX.—No. 474] AN EXTRA NURSING SUPPLEMENT. [Oct. 26, 1895.

### Sixty Thousand Pounds for the London Hospitals.

The year 1895 will be remembered as a red letter year in the records of the Hospital Sunday Fund. The present is the second occasion on which the newspaper Press of the metropolis has been the means of awakening life in the Hospital Sunday Fund movement and arousing interest in it amongst the citizens of London. Ten years ago, thanks to the late Dr. Wakley, the *Lancet* made special efforts to enforce an appeal for the hospitals, and the Fund was raised to £40,000. Since 1885-1886, when that appeal was made, the Fund has not shown much progress, but the average of £40,000 to £42,000 a-year has been maintained. This year, convinced of the fact that could Londoners be made to realise that if a sum of £70,000 were secured for the hospitals through the Sunday Fund, there need be no fear of want of funds, The Hospital, aided by generous citizens and the hearty co-operation of the metropolitan Press, made a special appeal which was brought to the notice of every well-to-do household in London. The Hospital Sunday Fund collections were made on June 15th, and the accounts will close on the 31st inst.

On Tuesday, in response to the appeal made in our "Special Hospital Sunday Supplement," the Lord Mayor received notice of a contribution from members of the Stock Exchange amounting to £3,400, which raised the collection this year to £50,000. On Thursday the Lord Mayor received a further sum of £15,000 for distribution amongst the charities, £10,000 of which was, in response to our appeal, to be added to the Hospital Sunday Fund. The sum at the disposal of the Distribution Committee of the Hospital Sunday Fund thus exceeds £60,000—a record collection full of hope for the future. Is it too much to hope that the splendid gifts we have now the pleasure to record may stimulate some wealthy persons and others who possess fair means, and are in a position to be generous, to send the remaining £9,000 or so to the Lord Mayor, at the Mansion House, during the next few days?

It may be profitable to consider for a moment the principle upon which the sum of upwards of £18,000, which has been sent to the Lord Mayor for the hospitals as the direct result of The Hospital's appeal of June 15th last, has been raised. The details, which will be found in another column, may be omitted here. It has been the fashion for many years past to preach eloquent sermons from the text that charity in this country is *in extremis.* We have never ceased to challenge this contention, and to prove by the production of facts and figures that it is not true so far as our great charities, and especially our best hospitals, are concerned. We have persistently urged that English men and women if properly approached are as willing to give to-day as ever before in the history of our country. To enforce this contention we have made a determined personal effort to help the hospitals this year through the Hospital Sunday Fund. The result is entirely satisfactory, and justifies the attitude we have taken up on this question of gifts to charities.

Two causes at present contribute to the confusion and despair of many excellent people who have to raise money for philanthropic objects. First, the absence of any means by which any individual, however unworthy, can be prevented from starting, entirely for his own private ends, what he audaciously describes as a hospital or as a charity of some other kind. Secondly, the growing habit amongst the managers of charities of issuing circular appeals through the post in increasing numbers. These two causes in practice often become identical. The adventurer not unnaturally displays little scruple and much astuteness. He issues a multitude of circular appeals and is, indeed, the originator of most of the ingenious methods brought to bear in the campaign of charity which is always proceeding in this country. We have a most interesting collection of literature of this kind, and have heard the adventurer maintain for thirty years past that it matters not to him, and it's no one else's business in the present state of the law, how much money he may expend to raise funds, provided he gets as much as he requires for himself. The adventurer has been known to spend £300 in advertisements and appeals for every £100 expended on his so-called charitable work. We are not surprised, therefore, to hear that where the appeals of the greater charities formerly produced large sums they now yield a comparatively miserable return. The managers ought to combine to move Parliament next session to empower the County Councils to make it impossible for adventure hospitals like the Jubilee Hospital to continue open unless radically reformed, and to prevent the establishment of any similar institutions in the future.

Meanwhile, the success of our efforts in connection with the Sunday Fund justifies the contention that the comparative failure of the appeal by circular is a hopeful sign for the best managed institutions. It proves that those who have the means of giving are beginning to think and act for themselves. There is probably no more laborious task than that which the rich man has to face every day, *i.e.*, the maintenance of his wealth. Unless he devotes his whole energies to the preservation and protection of his property, he may, and probably will, lose much or all of it. The world fails to realise the vastness of this burden of daily responsibility cast upon the rich man of to-day. His whole strength and energy may gradually become absorbed in the task, and life may ultimately have no pleasures for him except the promotion of schemes to increase his riches and so to add to the burden of his responsibility. Yet the rich men of England are as a class conscientious, able, industrious, and earnest people who have only to be convinced, to use the words of one of them recently dead, "that money can yield no pleasure equal to that of gifts to worthy objects of approved necessity," to become generous givers provided they can be guaranteed that the objects and charities they support are worthy and may properly be helped with money.

Our hope and desire is that the managers of our charities may reorganise their system of raising funds and place it largely upon a new and business basis. They will then take steps to supply the guarantees referred to, and so secure that the methods and objects of their work shall be brought home each year directly to the knowledge and consciences of an increasing number of persons who are able to give. Let them forsake the old methods of harassing appeals and humiliating entreaty, and let them preach from conviction the privilege of giving. The managers of charities would then become public benefactors in a double sense. Whilst affording succour to the poor, the needy, and the sick, they would provide those of the rich who have yet to learn to give liberally with an abiding source of happiness for the rest of their lives. Any who doubt the practical value of the views we express, must remember that in regard to upwards of £18,000 of the £60,000 constituting the record Hospital Sunday collection of 1895, the greater portion has been obtained without the expenditure of even a postage stamp. This fact affords striking proof of the wisdom and value of the principles we have here advocated.

